# Gestational Diabetes Mellitus and Incident Kidney Disease: A Nationwide French Cohort Study

**DOI:** 10.34067/KID.0000001068

**Published:** 2026-01-21

**Authors:** Justin B. Echouffo-Tcheugui, Solène Tapia, Sonia Bechraoui-Quantin, Jonathan Cottenet, Bernard G. Jaar, Emmanuel Simon, Catherine Quantin

**Affiliations:** 1 Division of Endocrinology, Diabetes and Metabolism, Department of Medicine, Johns Hopkins University, Baltimore, Maryland; 2 Welch Center for Prevention, Epidemiology, and Clinical Research, Department of Medicine, Johns Hopkins University, Baltimore, Maryland; 3 Biostatistics and Bioinformatics (DIM), University Hospital, Inserm, CIC, Dijon, France; 4 Dijon University Hospital, Clinical Investigation Center, Clinical Epidemiology/Clinical Trials Unit, Dijon, France; 5 Birth Center, Saint Francis Hospital, Colorado Springs, Colorado; 6 Department of Gynecology, Obstetrics, and Fetal Medicine, University Hospital of Dijon, Dijon, France; 7 Division of Nephrology, Department of Medicine, Johns Hopkins University School of Medicine, Baltimore, Maryland; 8 Université Paris-Saclay, UVSQ, Univ. Paris-Sud, Inserm, High-Dimensional Biostatistics for Drug Safety and Genomics, CESP, Villejuif, France

**Keywords:** AKI, CKD, diabetes mellitus

## Abstract

**Key Points:**

- In this large cohort, over 10 years, women with a history of gestational diabetes mellitus exhibited a high risk for overall kidney disease.
- This risk was already apparent in the early postpartum and more pronounced with two or more gestational diabetes mellitus episodes.
- Accounting for the history of gestational diabetes mellitus among women may help prevent kidney disease.

**Background:**

The extent to which gestational diabetes mellitus (GDM) influences the risk of kidney disease remains unknown. We investigated the associations between GDM and incidence of kidney disease, including CKD and AKI.

**Methods:**

This nationwide population-based cohort study included 1,441,317 parous women in France during 2012–2013. We used Cox regression to investigate the (*1*) association of GDM with incident hospitalization for AKI or CKD, (*2*) timing to postpartum GDM-related kidney disease, and (*3*) GDM recurrence and the incidence of kidney disease.

**Results:**

Over a 10-year period, women with a history of GDM (*n*=103,122 [7.2%]) had a 46% higher risk of CKD (adjusted hazard ratio [aHR], 1.46; 95% confidence interval [CI], 1.36 to 1.55) and a 18% higher risk of AKI (aHR, 1.18; 95% CI, 1.11 to 1.25), compared with those without a history of GDM. Accounting for postpartum incident hypertension and type 2 diabetes attenuated effect estimates—10% for CKD (aHR, 1.10; 95% CI, 1.02 to 1.19) and 1% for AKI (aHR, 1.01; 95% CI, 0.95 to 1.08). The elevated risk of kidney disease was apparent at 1 year postpartum and more pronounced among women with two or more GDM episodes than among those with one GDM episode.

**Conclusions:**

GDM was mainly associated with an increased risk of CKD, which is present early in the postpartum and higher among women with repeated GDM.

## Visual Abstract

**Figure:**
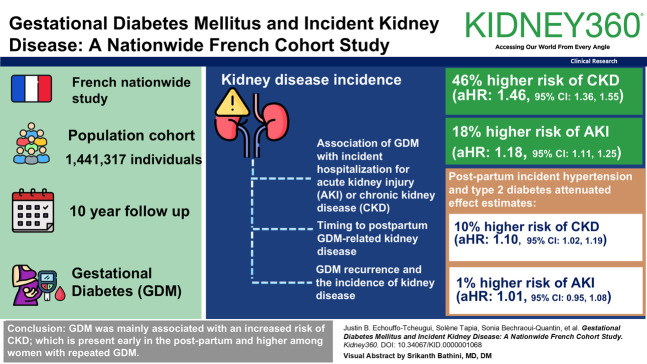

## Introduction

Gestational diabetes mellitus (GDM) has been increasingly common, affecting approximately 14% of pregnancy worldwide.^1^ Similarly, the frequency of kidney disease, especially that of CKD, is also increasing globally.^2^ GDM has been linked to a substantial burden of morbidity in the immediate and distant postpartum period, including a risk factors for kidney disease such as type 2 diabetes, hypertension, preeclampsia, and cardiovascular disease.^3–6^ However, the independent and direct links between GDM and kidney disease are less well described. Accruing evidence suggests that GDM is associated with a high risk of developing kidney disease, mainly CKD, in the years after pregnancy.^7–14^ However, there is a dearth of national studies on influence of GDM on the incidence of kidney disease.^7,10^ Moreover, most studies on the link between GDM and kidney disease have not accounted for the postpartum development of type 2 diabetes and hypertension and have not always examined AKI.^7–14^ The existing studies have also not specifically investigated the time to onset of kidney disease after GDM, which can importantly inform us on the appropriate window for early detection of renal disease and possibly an implementation of interventions to prevent progression in these young women. The published studies have also not examined the impact of GDM recurrence on kidney disease outcomes.^7–14^

Using a nationwide French cohort, we investigated the association of GDM with (*1*) incident hospitalization for kidney disease including AKI and CKD, (*2*) the timing to GDM-related kidney disease onset in the years after delivery, and (*3*) the effect of recurrent GDM on the risk of kidney disease. We hypothesized that the risk of kidney disease is elevated early in the postpartum period and rises overtime and also that the recurrence of GDM is associated with a much higher postpartum risk of kidney disease than a single episode of GDM.

## Methods

### Study Population

#### Data Source

Our sampling frame included all women in France with information in the French national Program de Médicalisation des Systèmes d’Information (PMSI), which is a medical administrative database that contains discharge abstracts from all public and private French hospitals. In PMSI, conditions diagnosed during a hospital stay are coded according to the International Classification of Diseases, 10th Revision (ICD-10), and procedures performed in hospitals are coded using the French Common Classification of Medical Procedures. The unique anonymous identification number generated for each patient capture in PMSI allows a linkage of all hospitalizations for an individual, thus allowing for longitudinal studies. The use of PMSI for budget allocation has fostered an improvement in accuracy and exhaustiveness of its data, which has been confirmed in internal and external validations. Consequently, the PMSI hospital data have been extensively used in medical research,^15,16^ and their high quality has been confirmed in perinatal studies.^17,18^ Almost all births are recorded in PMSI (out-of-hospital deliveries represent only 0.4% of births), which enables the coverage of all deliveries within a specified timeframe.^19^

#### Population

The present investigation includes all deliveries occurring during the period between January 1, 2012, and December 31, 2013. We used the ICD-10 code Z37 (except Z37.9) associated with different procedures to identify delivery (*N*=1,589,859). We excluded deliveries in women whose maternal age was <12 or >55 years to retain women of childbearing age (*N*=1,589,843 deliveries remaining) and then deliveries of women who died during the stay (*N*=1,589,728 deliveries remaining). We then excluded deliveries to women who had a history of diabetes mellitus (ICD-10 codes E10–E14) or GDM (ICD-10 code O24) before the index pregnancy—with a look back period of 5 years preceding the index pregnancy (*N*=1,551,303 deliveries remaining). We then excluded deliveries to women with a history of kidney diseases, especially CKD, identified using a look back period of 5 years before the index pregnancy (*N*=1,544,278 deliveries remaining). To count each woman only once, only the first pregnancy of the inclusion period and the associated delivery were considered (index pregnancy, *N*=1,514,837 women). To ensure a clean sample of women, we excluded women who did not have GDM at the index pregnancy (starting point of this study) but later developed GDM at a subsequent pregnancy during the whole study period (final sample of *N*=1,441,317 women). These women were categorized into women who had GDM during the inclusion period (*N*=103,122) and those who did not have GDM during either the inclusion period or the follow-up period (*N*=1,338,195). The process of selection of the study participants is shown in Supplemental Figure 1.

### Ethics Approval

This study was approved by the French National Committee for Data Protection (declaration of conformity to the methodology of reference 05 obtained on July 8, 2018, under the number 2204633 v0). Written informed consent was not needed for this study because this was a retrospective study and the national data used were anonymous.

### Ascertainment of GDM

We ascertained GDM at index pregnancy and subsequent pregnancies during the study period, among women without a history of diabetes mellitus, using the ICD-10 code O24 in the hospital (in-patient) records. In France, GDM is screened during in all hospitalizations during pregnancy.

### Kidney Outcomes

The kidney disease outcomes examined included AKI or CKD. The ICD-10 codes used for the identification of the AKI and CKD are presented in Supplemental Table 1. The codes for AKI included those for acute tubulointerstitial nephritis and unspecified acute renal failure. The codes used for identifying CKD included those for the followings: glomerular and proteinuria disease, chronic tubulointerstitial nephritis, unspecified CKD, hypertensive kidney disease, diabetes kidney disease, ESKD, renal dialysis, and kidney transplant.

The kidney disease events were ascertained from the hospital discharge records from the index delivery period to up to 10 years. If a woman was hospitalized for the same condition more than once during follow-up, only the first event was considered.

### Covariates

The covariates considered in our analyses included maternal age, vaginal delivery, smoking, alcohol use, morbid obesity (body mass index ≥40 kg/m^2^), and hypertensive disorders of pregnancy (including preeclampsia/eclampsia and gestational hypertension). The data on these covariates were obtained from all hospital stays during the index pregnancy period. We also examined preexisting chronic hypertension as well as postpartum hypertension and postpartum type 2 diabetes.

### Statistical Analyses

We examined the characteristics of the study population by GDM status, including all covariates but also parity, which is only available for vaginal deliveries in our database and thus could not be included in the regression models. We presented continuous variables as mean and SD (or median and interquartile range for variables with skewed distributions) and categorical variables as percentages. We tested the differences in baseline characteristics using chi-squared tests for categorical variables and appropriate parametric or nonparametric tests for continuous variables.

We derived the proportions of individuals developing the rates of kidney disease outcomes over the entire study period by GDM status. We assessed the risks of the occurrence of each kidney disease outcomes across the exposure categories (GDM versus no-GDM [reference]), using Cox proportional hazards regression models to derive hazard ratios (HRs) and 95% confidence intervals. The study participants who died during follow-up were censored, if the death was not related to kidney disease. In sensitivity analyses, we also used a Fine and Gray model to account for in-hospital mortality as a competing event. Thus, in this modeling, we followed women until their first hospitalization for the outcome, otherwise until their death, and otherwise until 10 years (administrative censoring time).

To assess the risk related to GDM recurrence, we performed Cox proportional hazard regression using the counting process formulation to consider the fact that another pregnancy with GDM may have occurred during follow-up. We also used the extended Cox regression with a step function model to estimate the effect of the exposure variable in different time intervals: before 1 year, from 1 to 3 years, from >3 to 5 years, and from >5 to 10 years of follow-up.^20,21^

All our models were adjusted for maternal age at index delivery, vaginal delivery, smoking, alcohol use, morbid obesity, hypertensive disorders of pregnancy (including preeclampsia/eclampsia and gestational hypertension), and preexisting chronic hypertension. We performed a first additional analysis, which further adjusted for the post-partum occurrence of chronic hypertension (as a time-dependent variable). We performed a second additional analysis, which further adjusted for the post-partum occurrence of type 2 diabetes (as a time-dependent variable).

Two-sided *P* values of <0.05 were considered statistically significant. All analyses were performed using SAS version 9.4 (SAS Institute, Cary, NC).

## Results

The study population consisted of 1,441,317 women (mean age 29.82±5.41 years), among whom 103,122 (7.2%) had GDM and 1,338,195 (92.8%) did not have GDM. The baseline characteristics of the study population by GDM status are presented in Table 1. Compared with women without a history of GDM, those with a history GDM were older, and had higher frequencies of smoking, alcohol use, morbid obesity, hypertensive disorders of pregnancy, and preexisting chronic hypertension. However, they had fewer vaginal deliveries and were more often multiparous that those without a history of GDM.

**Table 1:** Characteristics of women who gave birth in France between January 1, 2012, and December 31, 2013, according to gestational diabetes mellitus status

Characteristic	No GDM	GDM	*P* Value
	*N*=1,338,195	*N*=103,122	
Age, yr—mean (SD)	29.68 yr±5.37	31.71 yr±5.6	<0.0001
**Age groups, *N* (%)**			
<20 yr	35,496 (2.65)	996 (0.97)	<0.0001
20–29 yr	622,321 (46.50)	35,980 (34.89)	
30–39 yr	630,802 (47.14)	57,051 (55.32)	
≥40 yr	49,576 (3.70)	9095 (8.82)	
Vaginal delivery, *N* (%)	1,078,092 (80.56)	73,910 (71.67)	<0.0001
Multiparous (among vaginal deliveries), *N* (%)	604,698 (56.09)	45,099 (61.02)	<0.0001
Alcohol use, *N* (%)	714 (0.05)	105 (0.10)	<0.0001
Smoking, *N* (%)	56,914 (4.25)	5930 (5.75)	<0.0001
Morbid obesity, *N* (%)	5403 (0.40)	3128 (3.03)	<0.0001
Hypertensive disorder of pregnancy, *N* (%)	62,479 (4.67)	10,364 (10.05)	<0.0001
Preexisting chronic hypertension, *N* (%)	4451 (0.33)	1190 (1.5)	<0.0001
Postpartum hypertension, *N* (%)	12,897 (0.96)	3063 (2.97)	<0.0001
Postpartum type 2 diabetes, *N* (%)	2482 (0.19)	4898 (4.75)	<0.0001

GDM, gestational diabetes mellitus.

### GDM and Incident Kidney Outcomes

Over 10 years of follow-up, the cumulative unadjusted risk of kidney disease was higher among individuals with a history of GDM compared with those without such a history (Supplemental Table 2). Individuals with a history of GDM developed AKI (0.92% versus 1.10%, *P* < 0.001, unadjusted hazard ratio [HR], 1.19; 95% confidence interval [CI], 1.12 to 1.27) and CKD (0.63% versus 1.02%, *P* < 0.001, unadjusted HR, 1.62; 95% CI, 1.52 to 1.73) more often than those without a history of GDM.

In multivariable adjusted analyses, compared with individuals without GDM, the 10-year risks of overall kidney disease, CKD, and AKI were significantly higher in women with GDM compared with those without GDM (Table 2). Specifically, there was a 30% higher risk of CKD (adjusted hazard ratio [aHR], 1.46; 95% CI, 1.36 to 1.55), and a 18% higher risk of AKI (aHR, 1.18; 95% CI, 1.11 to 1.25) among women with a history of GDM compared with those without such history. In sensitivity analyses using the Fine and Gray model, probably given that the frequency of competing deaths was very low (0.16%), we obtained the same results as those obtained with the traditional Cox model.

**Table 2:** Associations of gestational diabetes mellitus with the hospital incidence of kidney disease over 10 years

Exposure	Adjusted HR (95% CI)	
	CKD	AKI
**GDM**
Base model^a^	1.46 (1.36 to 1.56)	1.18 (1.11 to 1.25)
Base model plus postpartum hypertension	1.35 (1.26 to 1.44)	1.12 (1.06 to 1.20)
Base model plus postpartum hypertension and postpartum type 2 diabetes	1.10 (1.02 to 1.19)	1.01 (0.95 to 1.08)
**GDM recurrence**
GDM during inclusion pregnancy only	1.41 (1.31 to 1.51)	1.11 (1.04 to 1.19)
GDM during at least two pregnancies	1.78 (1.53 to 2.07)	1.59 (1.38 to 1.83)

CI, confidence interval; GDM, gestational diabetes mellitus; HR, hazard ratio.

a Estimates of association are adjusted on maternal age, vaginal delivery, alcohol use, smoking morbid obesity, hypertensive disorders of pregnancy (preeclampsia/eclampsia or gestational hypertension), and chronic hypertension (pre-existing).

In a first additional analysis that accounted for the occurrence of chronic hypertension during postpartum follow-up as time-dependent variable, we observed a slight reduction in the effect estimates for each of the renal outcomes—aHR, 1.35 (95% CI, 1.26 to 1.44) for CKD and aHR, 1.12 (95% CI, 1.06 to 1.20) for AKI. In the second additional analysis, we further adjusted for of postpartum incident type 2 diabetes, which further attenuated the magnitude of the effect estimates—10% higher risk of CKD (aHR, 1.10; 95% CI, 1.02 to 1.19) and 1% higher risk for AKI (aHR, 1.01; 95% CI, 0.95 to 1.08).

### GDM Recurrence and Incident Kidney Disease

The risks for kidney disease events associated with at least two pregnancies with GDM were significantly higher than the corresponding risks associated with a single pregnancy with GDM, when compared with no pregnancy with GDM (Table 2).

The corresponding risks estimates for two pregnancies or more were 78% (aHR, 1.78; 95% CI, 1.53 to 2.07) for CKD and 59% for AKI (aHR, 1.59; 95% CI, 1.38 to 1.83). The corresponding risks for having had GDM at one pregnancy only were 41% and 11% for CKD and AKI, respectively.

### Time to Onset of Kidney Disease

The examination of the influence of GDM at different time intervals (during the first year, from 1 to 3 years, from >3 to 5 years, and from >5 to 10 years of follow-up) showed a steady increase in the risks of CKD and AKI associated with GDM over time, with the heightening of the risk observed as early as from the first year after delivery (Table 3). Specifically, for CKD, we observed a 29% higher risk (aHR, 1.29; 95% CI, 1.05 to 1.58) in the first year, which steadily increased over subsequent time intervals reaching 51% in the 5–10-year period (aHR, 1.51; 95% CI, 1.38 to 1.65).

**Table 3:** Time intervals^a^ adjusted^b^ associations of gestational diabetes mellitus with the hospital incidence of kidney diseases over 10 years

Time Interval	Adjusted HR (95% CI)	
	CKD	AKI
Before 1 yr	1.29 (1.05 to 1.58)	1.04 (0.86 to 1.24)
1–3 yr	1.38 (1.19 to 1.60)	1.05 (0.91 to 1.20)
3–5 yr	1.51 (1.31 to 1.74)	1.09 (0.95 to 1.25)
5–10 yr	1.51 (1.38 to 1.65)	1.33 (1.22 to 1.46)

CI, confidence interval; HR, hazard ratio.

a Extended Cox regression with step function multivariable model.

b Adjusted on maternal age, vaginal delivery, smoking, alcohol use, morbid obesity, hypertensive disorders of pregnancy (preeclampsia/eclampsia or gestational hypertension), and chronic hypertension.

## Discussion

In a nationwide cohort of French women followed over a 10-year period after delivery, a history of GDM was associated with increased risks of kidney disease, mainly CKD, independent of pregnancy-related factors (including hypertensive disorders of pregnancy), and chronic hypertension. These risks were significantly attenuated when postpartum development of hypertension and type 2 diabetes were accounted for, remaining significant for CKD, but not for AKI. The risk of kidney disease was much higher in women who had experienced a recurrence of GDM than among those with a single GDM episode. The elevated risk of kidney disease was also evident as early as 1 year after the index pregnancy and increased over time.

To the best of our knowledge, our study is the largest nationwide study to evaluate the effect of GDM on the postpartum risk of kidney disease in France, based on a combination of perinatal and postpartum data. Our study provides additional and valuable evidence on the links between GDM and kidney disease. Our results are largely congruent with those of the vast majority of investigations showing an increased risk of kidney disease in relation to GDM.^7–14^ Of note, previous studies mainly examined CKD, not including AKI in their investigations. Our results are comparable with those of an equivalent nationwide Danish cohort, which showed increased in the risks of AKI and CKD associated with GDM. In addition to being to date the largest investigation on the association of GDM and kidney disease, our study examined aspects previously unexplored, and makes a unique contribution to our understanding of the association between GDM and CKD. Indeed, our study provides novel evidence on: (*1*) the risk of postpartum kidney disease associated with a recurrence of GDM and (*2*) the timing to onset of kidney disease and the trajectory of risk over time. Our results on a higher risk of kidney disease associated with two or more pregnancies as compared with only one pregnancy with GDM, suggest a monotonic association between the number of GDM episode and the postpartum risk for kidney disease. A finding that supports the possible causal nature of the GDM and kidney disease associations.^22^

Our results have clinical potential implications for postpartum health monitoring in pregnancies. These suggest that a monitoring of renal function in the aftermath of pregnancies complicated by GDM in young women may be warranted. Moreover, our results on the timing of GDM-related kidney disease support an early initiation of such a surveillance. This is important as testing for renal function as part of the immediate or distant post-partum monitoring after GDM is not formally recommended in the current guidelines. The potential relevance of kidney disease monitoring in the postpartum period is further supported by the rising burden of kidney disease, especially CKD, in France and globally.^23^ Given that most women (>80%) will become pregnancy during their lifetime,^24^ the occurrence of GDM and its aftermath offers a unique window of opportunity for preventing renal disease among young women.

The mechanistic pathways linking GDM to kidney disease are still to be clarified. The of GDM and kidney disease association may be mediated by the effects of GDM on precursors of kidney disease such as hypertension, type 2 diabetes, and preeclampsia. However, the persistence of the association after accounting for these factors suggests an intrinsic GDM effect on the kidney. Among other processes that may link GDM to kidney disease is insulin resistance,^25^ which is a prominent and shared feature between GDM and CKD.^26,27^ Other processes similar to those observed in the context of diabetic kidney disease may also be at play in the setting of GDM, including glomerular basement member and mesangial changes, podocyte injury, inflammation, and metabolic memory.^28^

There are some limitations to our study. First, although there is universal access to free health care in France, the uptake of GDM screening will not have been 100%; hence, there were some women with undiagnosed GDM in the reference group, contributing to likely underestimation of risks associated with GDM. Moreover, the classification of postpartum diabetes status was limited to known diagnoses and not based on any glycemic measures. Second, the identification of kidney disease was solely based on hospital diagnosis codes (not including laboratory-based data on eGFR and/or albuminuria), which will inevitably lead to an underestimation of the incidence of kidney disease. The diagnosis of CKD is more likely to have been documented for the later stages of CKD than earlier stage, given the clinical relevance of the later CKD stages for a hospital admission. Indeed, a number of women with CKD will only have microalbuminuria or reduced eGFR >60 ml/min per 1.73 m^2^ and may not be referred to the hospital and thus may be missed. Third, our definition of GDM was not based on blood glucose test results but on codes from PMSI administrative data. However, this approach has been shown to be a reliable and valid method of ascertainment.^29^ Fourth, we lacked data on the use of medications in the postpartum period that may have a protective effect on renal function. Fifth, the generalizability of our findings is potentially restricted to populations similar to the French population with a similar racial/ethnic make and underlying distribution of risk factors for kidney, as well as similar health care services coverage. Finally, there is possibility of residual confounding, especially as some risk factors are not included in our adjustments, including for example recurrent infections, family history of kidney disease, use of medications that can affect kidney outcomes, and lifestyle factors.

The strengths of our study are manifold. First, our study is a large prospective registry-based cohort study including almost all pregnant women in France,^19^ from a health system in which GDM is systematically assessed in pregnant women, thus minimizing potential bias including selection bias, referral, and recall bias. Second, the accurate data linkage allowed for the examination of a wide range of kidney disease outcomes over a long enough follow-up period. Although misclassification is not completely ruled out, the ascertainment of kidney disease outcomes using ICD codes in administrative databases has been shown to be reliable and valid.^30,31^ Third, we accounted for numerous confounders including pregnancy-related and non–pregnancy related factors, and our results were robust to sensitivity analyses accounting for the postpartum development of type 2 diabetes and hypertension.

In conclusion, we showed that a history of GDM was significantly and independently associated with increased risks of kidney disease, specifically CKD, among French women. This rise in kidney disease risk was evident in the early postpartum period and increased with time. The risk of kidney disease was also pronounced among women with a recurrence of GDM. These results suggest continuous kidney function monitoring among women with a history of GDM in the postpartum, especially among those with recurrent GDM, may help prevent the onset and progression of kidney disease.

## Supplementary Material

## Data Availability

Original data generated for the study will be made available upon reasonable request to the corresponding author. Data Type: Observational Data. Reason for Restricted Access: The data used in this study were retrieved from the French program—PMSI—and do not belong to the authors French Government. The authors are therefore not permitted to make the data publicly available. However, upon receipt of the necessary approvals from the PSMI, data can be made available.

## Declarative Statements

This study includes clinical experimentation and received Institutional Review Board or Ethics Committee approval. The need to obtain informed patient consent was waived.

## Supplemental Material

Supplemental Figure 1. Study flow chart.

Supplemental Table 1. ICD-10 codes used to identify the study outcomes and covariates.

Supplemental Table 2. Prevalence and unadjusted risk (Cox model) of kidney disease among women with and without GDM in the 10 years following the index delivery.
