# Supplementary material for: Gestational Diabetes Mellitus and Incident Kidney Disease: A Nationwide French Cohort Study
Source: Kidney360. 2026 Jan 21;7(7):1556–62. doi: 10.34067/KID.0000001068 (PMC13450958; doi:10.34067/KID.0000001068)
Supplement: Supplementary file 1 [file kidney360-7-1556-s001.pdf]

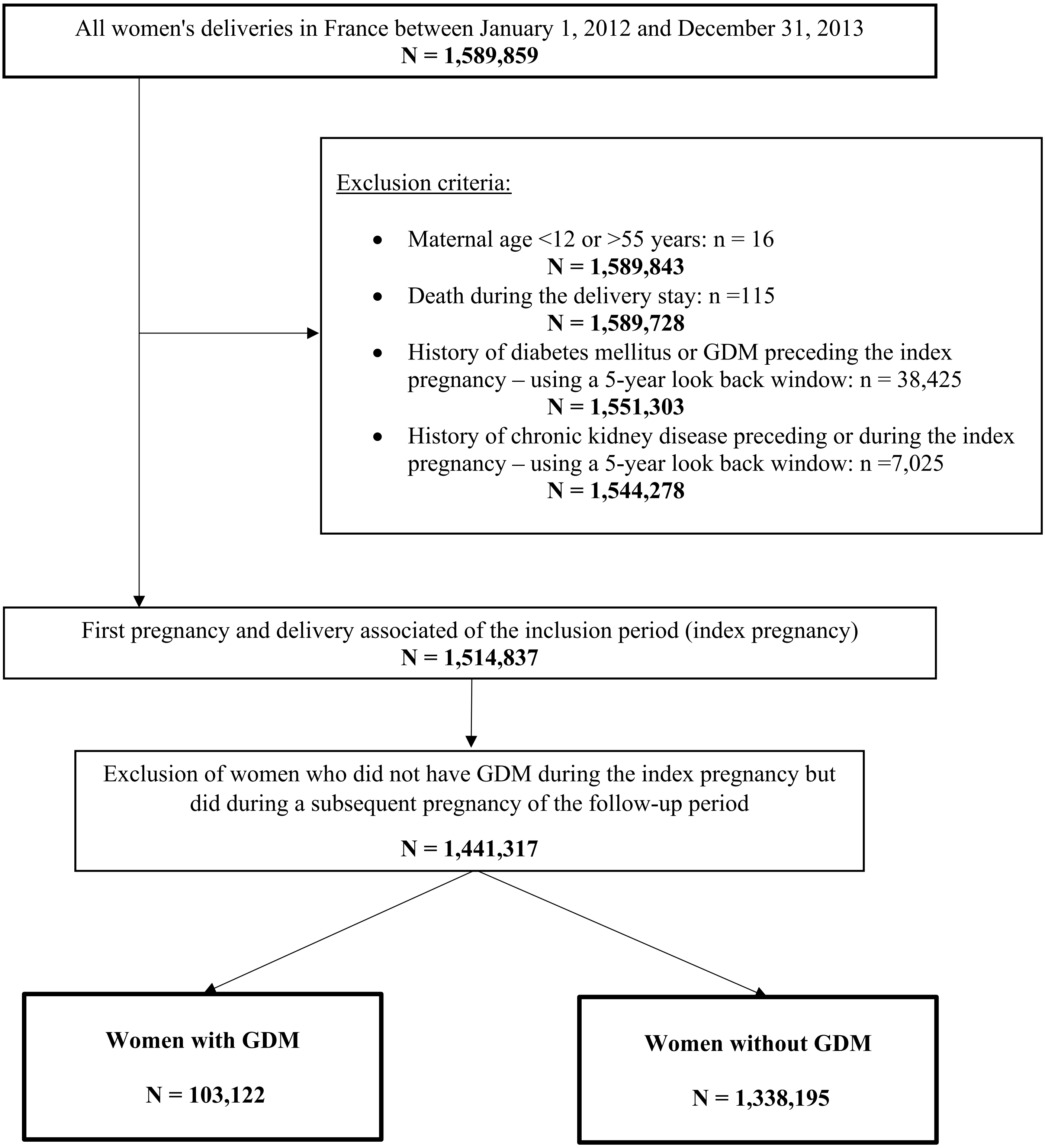

Supplemental Figure 1: Study Flow chart

GDM: gestational diabetes.

Supplemental Table 1. ICD-10 codes used to identify the study outcomes and covariates

	ICD 10 codes
**Chronic kidney disease (CKD)**	
Chronic kidney disease (CKD)	N01-N08, N11-N13, N15-N16, N18-N19, I12-I151, Z49, Z992, Z905, Z940, T861, E102, E112, E132
Glomerular and proteinuric disease	N01-N08, except N083
Chronic tubulointerstitial nephritis	N11-N12, N15-N16
Unspecified CKD	N18-N19
Hypertensive kidney disease	I12-I151
Diabetes kidney disease	E102, E112, E132, N083
Other unspecified CKD	N13, N18-N19, Z49, Z992, Z940, T861
ESRD	N185, Z992, Z940, T861
Renal dialysis	Z49, Z992
Kidney transplant	Z905, Z940, T861
**Acute Kidney Injury (AKI)**	
Acute kidney injury (AKI)	N10, N17
Acute tubulointerstitial nephritis	N10
Unspecified acute renal failure	N17
**Covariates**	
Smoking	F17 (except F17.0), T65.2 and Z72.0
Alcohol use	F10 (except F10.0), G62.1, G72.1, I42.6, K29.2, K85.2, K86.0 and K70
Morbid obesity (BMI≥40 kg/m2)	E6601-02, E6606-07, E6611-12, E6616-17, E6621-22, E6626-27, E6681-82, E6686-87, E6691-92 and E6696-97
Hypertensive disorder of pregnancy (including preeclampsia/eclampsia and gestational hypertension)	O10-O16
Chronic hypertension	I10-I15
Type 2 diabetes	E10 -14

Supplemental Table 2. Prevalence and unadjusted risk (Cox model) of kidney disease among women with and without gestational diabetes (GDM) in the 10 years following the index delivery

Outcomes	No GDM*		GDM*		p-value	Unadjusted Hazard Ratio (95% Confidence Interval)
	N = 1,338,195 (92.8%)		N = 103,122 (7.2%)			
	n	%	n	%		
Chronic kidney disease (CKD)	8,441	0.63	1,050	1.02	<.0001	1.62 (1.52, 1.73)
Glomerular and proteinuric disease	865	0.06	128	0.12	<.0001	1.92 (1.60, 2.31)
Chronic tubulointerstitial nephritis	2,408	0.18	249	0.24	<.0001	1.34 (1.18, 1.53)
Unspecified CKD	1,006	0.08	130	0.13	<.0001	1.68 (1.40, 2.01)
Hypertensive kidney disease	275	0.02	34	0.03	0.0087	1.61 (1.12, 2.29)
Diabetes kidney disease	67	0.01	194	0.19	<.0001	37.62 (28.50, 49.66)
Other unspecified CKD	5,490	0.41	575	0.56	<.0001	1.36 (1.25, 1.48)
ESRD	310	0.02	25	0.02	0.8269	1.05 (0.70, 1.57)
Renal dialysis	260	0.02	22	0.02	0.6735	1.10 (0.71, 1.70)
Kidney transplant	379	0.03	35	0.03	0.3049	1.20 (0.85, 1.70)
Acute kidney injury	12,341	0.92	1,135	1.10	<.0001	1.19 (1.12, 1.27)
Acute tubulointerstitial nephritis	10,956	0.82	964	0.93	<.0001	1.14 (1.07, 1.22)
Unspecified acute renal failure	1,659	0.12	205	0.20	<.0001	1.60 (1.39, 1.86)

GDM : Gestational Diabetes Mellitus
